# Smooth-muscle myosin mutations in hereditary non-polyposis colorectal cancer syndrome

**DOI:** 10.1038/sj.bjc.6604737

**Published:** 2008-10-21

**Authors:** N Vickaryous, G Polanco-Echeverry, S Morrow, N Suraweera, H Thomas, I Tomlinson, A Silver

**Affiliations:** 1 Colorectal Cancer Genetics, Institute for Cell and Molecular Sciences, Barts and The London School of Medicine and Dentistry, Queen Mary University of London, Blizard Building, 4 Newark St, Whitechapel, London E1 2AT, UK; 2 Family Cancer Clinic, St Mark's Hospital, Harrow HA1 3UJ, UK; 3 Molecular and Population Genetics Laboratory, London Research Institute, Cancer Research UK, London WC2A 3PX, UK

**Keywords:** hereditary non-polyposis colorectal cancer, microsatellite instability, smooth-muscle myosin

## Abstract

We examined adenomas and cancers from hereditary non-polyposis colorectal cancer (HNPCC) syndrome patients for the presence of frameshift mutations in the smooth-muscle myosin gene, *MYH11*. Our results show that mutations in *MYH11* occur more frequently in cancers than adenomas (*P*=0.008) and are dependent on microsatellite instability (MSI+).

DNA microsatellite instability (MSI+) is a hallmark of tumours where there is loss of a mismatch repair (MMR) protein. This is exemplified in patients with hereditary non-polyposis colorectal cancer (HNPCC), an autosomal-dominant syndrome defined by predisposition to colorectal cancer (CRC; 2–5% of all incident cases) and to other cancers, including endometrial, stomach and ovarian cancer. Genetically, HNPCC is characterised by germline mutation and somatic inactivation of the MMR genes (Liu *et al*, 1996; Lynch and Smyrk 1996; Lagerstedt Robinson *et al*, 2007). MSI+ CRCs also account for approximately 15% of all sporadic cases. Microsatellites have been characterised as being prone to frameshift mutations in MSI+ cancers and mutations in coding regions of genes such as *TGFβRII*, *BAX*, and *MSH6*, have been implicated in contributing to the neoplasia phenotype (Johannsdottir *et al*, 2000).

The genetic pathways of colorectal tumorigenesis in HNPCC and sporadic MSI+ CRCs may overlap. Indeed, much of what we know about HNPCC has been conflated with that of the more common sporadic MSI+ CRCs. However, there are a number of important differences, particularly with regard to *K-RAS*, *BRAF*, and *β-*catenin mutational frequencies (Johnson *et al*, 2005a, 2005b; Loughrey *et al*, 2007). Recently, the SM2 isoform of the smooth-muscle myosin gene, (*MYH11*) was implicated in human intestinal neoplasia. Somatic frameshift mutations at a coding region containing a repeat of eight cytosines, (C)_8_, in the final exon of *MYH11* were identified in sporadic MSI+ cancers and a germline frameshift mutation was also described in a Peutz–Jeghers syndrome patient (Alhopuro *et al*, 2008). To establish the contribution, timing and frequency of the *MYH11* frameshift mutation in HNPCC tumorigenesis, we analysed a series of adenomas and cancers from HNPCC patients.

## Materials and methods

### Patients

Hereditary non-polyposis colorectal cancer was diagnosed based on previously reported criteria and national ethics guidelines were followed (Johnson *et al*, 2005a, 2005b). A total of 77 HNPCC individuals from 60 families were selected and 34 adenomas plus 53 CRCs collected. Cancers were obtained from the distal and proximal colon and all grade from poor to well-differentiated and Duke stages A–C were represented as well as tumours that had metastasised to distant sites (D). Four endometrial cancers were also investigated. Hereditary non-polyposis colorectal cancer matched normal tissue was available for 41 samples.

### Mutation analysis

DNA was prepared by dissection of neoplastic and normal areas from paraffin-embedded tissue followed by a proteinase K digestion. MSI status was defined using microsatellite markers *BAT-25*, *BAT-26*, *D2S123*, *D5S346*, and *D17S250* analysed on an ABI-3100 genetic analyzer and classified as MSI+ as per Bethesda guidelines (Boland *et al*, 1998).

The *MYH11* mutation was examined using labelled primers flanking the (C)_8_ tract (accession no. NM_022844; nucleotide nos. 5898–5905) and analysed on an ABI-3100 genetic analyser. Detected mutations were verified using direct sequencing. Frequency of frameshift mutations at intronic (C)_8_ tracts were determined with primer pairs flanking regions in *DHX30*, *DDELF1*, *ILIR2* and *PAK3* genes (Alhopuro *et al*, 2008). Primer details are available on request.

### Statistics

Fisher's exact test was used to test for significance using Stata 8.2 (statistical software release 8.0; Stata corporation College Station, TX, USA).

## Results and discussion

The (C)_8_ repeat tract in the last exon of the SM2 isoform of *MYH11* was examined in 87 HNPCC intestinal tumours. MSI+ adenomas had a mutation rate of 5% (1/20) with a single adenoma carrying a frameshift mutation in one allele (Table 1). In contrast to MSI+ adenomas, MSI+ cancers had a much higher rate of frameshift mutation (36%, 17 out of 47; *P*=0.008; Table 1). There were fewer mutations in our HNPCC MSI+ cancers (36%) compared with the sporadic MSI+ cancers (55%) reported by Alhopuro *et al* (2008) and this difference was significant (*P*=0.04). We also investigated four MSI+ endometrial cancers and found *MYH11* frameshift mutations in 50% of samples (2 out of 4; Table 1). No frameshift mutations were found in the matched normal DNA (*n*=41) from HNPCC patients, confirming the somatic origin of mutations. There were also no mutations found in microsatellite stable (MSI−) adenomas (*n*=14) or MSI− cancers (*n*=6; Table 1).

A concern when analysing allele mutation frequency in MSI+ tumours is the confounding issue of passenger mutations that do not contribute to neoplasia. We assessed mutation frequency at four loci containing intronic (C)_8_ repeat tracts in which any frameshift mutation would not be predicted to be selected for and would therefore represent passenger mutations (Alhopuro *et al*, 2008). We found that mutation frequencies at the intronic (C)_8_ repeats were higher in HNPCC MSI+ cancers (16% of all loci, 29 out of 179) as compared with MSI+ adenomas (7% of all loci, 5 out of 69; *P*=0.07) indicating increased passenger mutation rates in the MSI+ CRCs. More importantly, MSI+ cancers had significantly higher frameshift mutation rates in *MYH11* than at the intronic repeats (36 *vs* 16%, *P*=0.004). MSI+ adenomas however, displayed no difference between frequency of *MYH11* (5%, 1 out of 20) and intronic (C)_8_ frameshift mutations (7%, 5 out of 69; *P*=1.0) These results indicate that frameshift mutations at *MYH11* do not play a role in early stages of tumour formation, but are likely to play a role in progression of HNPCC tumorigenesis.

We next investigated whether there were associations between the presence of an *MYH11* mutation and pathological features such as Dukes stage, tumour grade and tumour site. Dukes stage was available for 33 cancers (Dukes A+B, *n*=21; Dukes C+D, *n*=12) and frameshift mutations were found in 29% (6 out of 21) of the A+Bs and 25% (3 out of 12) of the C+Ds indicating that mutation frequency was not increased in the more advanced Dukes stage (*P*=1.0; Table 2). A total of 29 cancers had tumour grade available and although the numbers in the well- and poorly differentiated grades were small, there was a trend towards an increase in mutation frequency with grade (well differentiated, 0%, 0 out of 3; moderately, 27%, 6 out of 22; poorly differentiated, 50%, 2 out of 4; Table 2), but this was not significant (*P*=0.4). Mutation frequency was not influenced by site of tumour presentation, distal or proximal (*P*=1.0; Table 2).

In conclusion, our results indicate that the *MYH11* mutation is not required for early HNPCC adenoma formation, but it is selected for in the process of MSI+ cancer tumorigenesis. The role of *MYH11* in the development of MSI+ cancers merits further investigation particularly with respect to the underlying molecular and cellular mechanism.

## Figures and Tables

**Table 1:** Frequency of *MYH11* frameshift mutations in HNPCC tumours

	**C8/C8^a^**	**C7/C8**	**C8/C9**	**C7/C8/C9^b^**	**Mutation rate**
Normal	41	0	0	0	0/41 (0%)
MSI− adenoma	14	0	0	0	0/14 (0%)
MSI− cancer	6	0	0	0	0/6 (0%)
MSI+ adenoma	19	1	0	0	1/20 (5%)
MSI+ cancer	30	13	2	2	17/47 (36%)
Endometrial cancer	2	2	0	0	2/4 (50%)

a Wild type alleles.

b Indicates tumour heterogeneity.

**Table 2:** Pathological features of HNPCC tumours and frequency of *MYH11* mutation

	**Number of samples**	**Number without mutation**	**Number with mutation**	**Mutation frequency**	***P*-value**
*Dukes stage* a					
A+B	21	15	6	29%	
C+D	12	9	3	25%	1.0
Total	33	24	9		
					
*Tumour grade*					
Well differentiated	3	3	0	0%	
Moderately differentiated	22	16	6	27%	
Poorly differentiated	4	2	2	50%	0.4
Total	29	21	8		
					
*Tumour site*					
Proximal location	13	9	4	31%	
Distal location	12	9	3	25%	1.0
Total	25	15	7		

a Dukes stages A–C and tumours that had metastasised to distant sites (D).
